# Aberrant post-translational modifications compromise human myosin motor function in old age

**DOI:** 10.1111/acel.12307

**Published:** 2015-02-02

**Authors:** Meishan Li, Hannah Ogilvie, Julien Ochala, Konstantin Artemenko, Hiroyuki Iwamoto, Naoto Yagi, Jonas Bergquist, Lars Larsson

**Affiliations:** 1 Department of Physiology and Pharmacology, Karolinska InstitutetSE-171 77, Stockholm, Sweden; 2 Department of Clinical Neuroscience, Clinical Neurophysiology, Karolinska InstitutetSE-171 77, Stockholm, Sweden; 3 Centre of Human and Aerospace Physiological Sciences, Faculty of Life Sciences and Medicine, King's College LondonLondon, SE1 1UL, UK; 4 Analytical Chemistry, Department of Chemistry – Biomedical Centre and SciLifeLab, Uppsala University75124, Uppsala, Sweden; 5 Japan Synchrotron Radiation Research Institute, SPring-8Hyogo, 679-5198, Japan

**Keywords:** aging, function, myosin, post-translational modification, skeletal muscle

## Abstract

Novel experimental methods, including a modified single fiber *in vitro* motility assay, X-ray diffraction experiments, and mass spectrometry analyses, have been performed to unravel the molecular events underlying the aging-related impairment in human skeletal muscle function at the motor protein level. The effects of old age on the function of specific myosin isoforms extracted from single human muscle fiber segments, demonstrated a significant slowing of motility speed (*P *<* *0.001) in old age in both type I and IIa myosin heavy chain (MyHC) isoforms. The force-generating capacity of the type I and IIa MyHC isoforms was, on the other hand, not affected by old age. Similar effects were also observed when the myosin molecules extracted from muscle fibers were exposed to oxidative stress. X-ray diffraction experiments did not show any myofilament lattice spacing changes, but unraveled a more disordered filament organization in old age as shown by the greater widths of the 1, 0 equatorial reflections. Mass spectrometry (MS) analyses revealed eight age-specific myosin post-translational modifications (PTMs), in which two were located in the motor domain (carbonylation of Pro79 and Asn81) and six in the tail region (carbonylation of Asp900, Asp904, and Arg908; methylation of Glu1166; deamidation of Gln1164 and Asn1168). However, PTMs in the motor domain were only observed in the IIx MyHC isoform, suggesting PTMs in the rod region contributed to the observed disordering of myosin filaments and the slowing of motility speed. Hence, interventions that would specifically target these PTMs are warranted to reverse myosin dysfunction in old age.

## Introduction

Falls are a major cause of morbidity and mortality in the growing population of elderly citizens, constituting the leading reason of unintentional injury deaths in individuals over the age of 79. The source of falls and fall-related injuries in old age are complex and involve multiple risk factors. The aging-related impairments in somatosensation, vision, and vestibular function have negative effects on maintaining postural balance. However, the difficulty in recovering from a threatening fall, which is impaired in old age, is not related to an impairment of the sensory process or to the motor planning that leads to the initiation of muscle contraction (Schultz *et al*., 1997). Events after depolarization of skeletal muscle play a major role in the inability to correct the fall, that is, in force-generating capacity and contractile speed (Schultz *et al*., 1997). In support of this, the decline in mobility and lower extremity disability has been reported most influential in predicting falls in the elderly (Whipple *et al*., 1987; Blake *et al*., 1988; Robbins *et al*., 1989). Further, aging affects specific types of muscles, motor units, and muscle cells differently, and a detailed understanding of sarcopenia requires studies of muscle fibers of both the fast- and slow-twitch type (Larsson & Ansved, 1995). Hence, understanding the aging muscle, more specifically the loss of speed and force in old age, appears primordial in deciphering potential ‘preventative’ or ‘therapeutic’ approaches.

Muscle contraction requires the proper functioning of the sarcomere, and thus, one of its major structural components, the motor protein myosin. Myosin molecules have a slow turnover rate, that is, 1–2% per day (Smith & Rennie, 1996) which makes them preferential targets for different types of post-translational modifications (PTMs) by reactive oxygen or nitrogen species. We have previously shown that there is evidence to indicate that PTMs may affect enzymatic activity, stability, and digestibility of myosin during aging (Larsson & Ansved, 1995; Hook *et al*., 1999). A few studies have tried to quantify PTMs in myosin molecules by focusing on a few selected markers, including carbonylation, nitration, formation of HNE (4-hydroxy-2-nonenal) adducts, and glycation (Syrovy & Hodny, 1992; Watanabe *et al*., 1992; Oh-Ishi *et al*., 2003). Unfortunately, there is no consensus regarding aging-related changes in the levels of these markers or their precise localization. Indeed, oxidation of one or two reactive myosin cysteines (Cys707 and Cys696) could be crucial and may well trigger motor dysfunction (Crowder & Cooke, 1984). Similarly, glycation of some myosin lysine-rich regions that are important for actin binding and ATPase activity may play a primordial role (Ramamurthy *et al*., 2001; Ramamurthy & Larsson, 2013).

In this study, we aimed to determine the nature and location of PTM sites using mass spectrometry in parallel with assessment of the catalytic and force-generating capacity of specific myosin isoforms using a modified single fiber *in vitro* motility assay and measure conformational changes during single muscle fiber contraction using small-angle X-ray scattering. We initially hypothesized that aging-specific carbonylations, nitrations, and/or glycations in the myosin motor domain would compromise the myosin head binding to actin filaments subsequently resulting in a decreased speed of contraction and force production.

## Results

### Aging effects on the function of myosin isoforms

All *in vitro* motility measurements have been restricted to muscle fibers expressing the type I and type IIa MyHC isoform and fulfilling the criteria for acceptance, due to the paucity of muscle fibers expressing the IIx MyHC isoform in both young and old subjects. Results from *in vitro* motility measurements in fibers expressing the same MyHC isoform have been pooled as there was no difference between individuals within the young and the old age groups. A total of nine and ten fibers from the young subjects, 11 and 15 fibers from the old subjects expressing the type I and IIa MyHC isoforms, respectively, were included in the analyses. Single fibers were obtained from three young and five old subjects. Regarding the evaluation of myosin under the oxidative stress *in vitro*, a total of seven fibers were collected from one additional young male subject and myosin function was compared between one half exposed to oxidative stress and the other half of the fiber serving as control.

**Figure 1:**
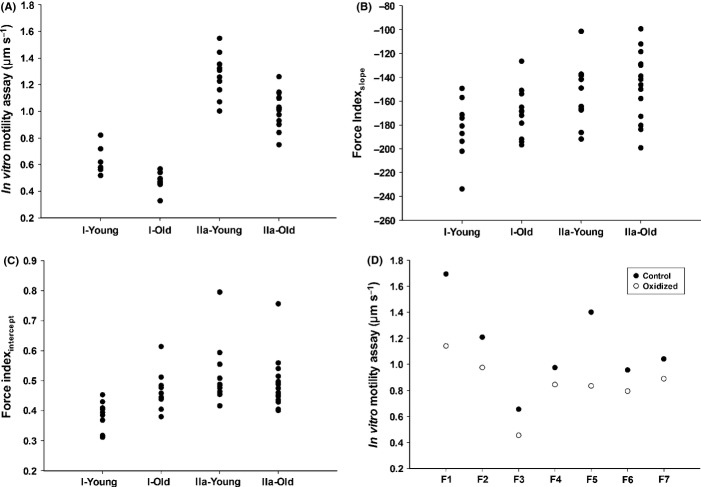
*Myosin function* The distribution of motility speed (A), force index_slope_ (B), and force index_intercept_ (C) of MyHC isoforms (type I and IIa) in young and old human muscle. The motility speed of the two halves of seven different single fibers (the oxidized and the control) (D).

The motility speed was highly dependent on MyHC isoform as proven by the absence of overlap between data from type I and IIa MyHC isoforms. This strong dependence on MyHC isoform expression was present in both age groups (Fig.1A). However, an aging-related slowing of motility speed was observed in both type I and IIa MyHC assays, that is, motility speed declined from 0.62 ± 0.09 μm s^−1^ to 0.47 ± 0.06 μm s^−1^ in type I MyHC (*P *<* *0.001) and from 1.27 ± 0.17 μm s^−1^ to 1.02 ± 0.13 μm s^−1^ in type IIa MyHC (*P *<* *0.001) assays (Fig.1A).

The force index measured both as the slope and *x*-axis intercept showed a large variability within each MyHC preparation and demonstrated a significant overlap between type I and IIa MyHC isoforms, but both the force index_slope_ (*P *<* *0.05) and force index_intercept_ (*P *<* *0.01) showed a higher force index in type IIa than type I; however, there was no significant aging-related change in the force indices (Fig.1B,C). In the type I MyHC, force index_slope_ was −183 ± 25 vs. −170 ± 21 and force index_intercept_ was 0.39 ± 0.05 vs. 0.46 ± 0.06 in young and old subjects, respectively. In the type IIa MyHC, force index_slope_ was −154 ± 27 vs. −146 ± 28 and force index_intercept_ was 0.52 ± 0.11 vs. 0.49 ± 0.09 in young and old subjects, respectively.

The motility speed of myosin molecules extracted from hydrogen peroxide treated fibers was significantly decreased (*P *<* *0.05) from 1.04 to 0.84 μm s^−1^ (Fig.1D). Similar to the comparisons between young and old subjects, both the force index_slope_ (*P *=* *0.536) and force index_intercept_ (*P *=* *0.256) were not significantly different, that is, force index_slope_ were −155 ± 40 vs. −138 ± 46 and force index_intercept_ were 0.55 ± 0.09 vs. 0.63 ± 0.13 in the control and oxidized muscle fibers, respectively.

### Myosin molecules are disordered during contraction in old age

**Figure 2:**
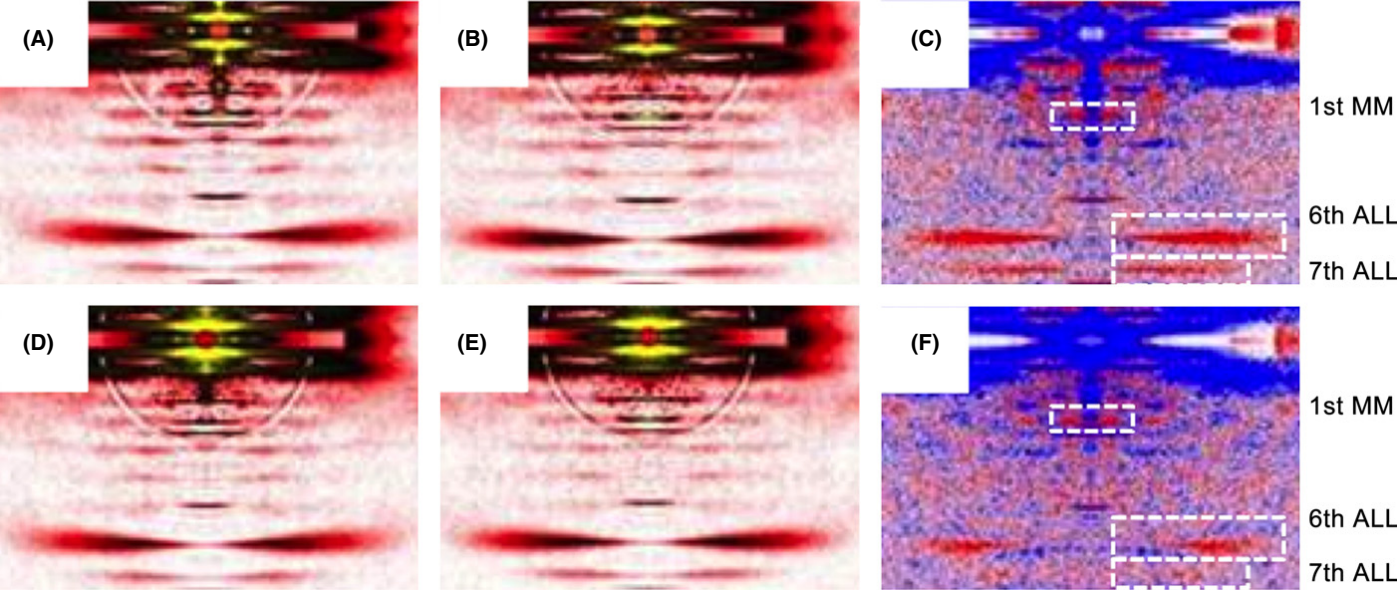
*X-ray diffraction patterns* Low angle X-ray diffraction reflections. X-ray diffraction patterns of fibers from young (first row: A, B, and C) and older individuals (second row: D, E, and F), in pre-activating (pCa 9.0, first column: A and D) and activating (pCa 4.5, second column: B and E) solutions. There are also differences in intensity profiles (third column: C and F); red and blue colors indicate the enhanced and weakened areas, respectively, after addition of calcium. MM, myosin meridional; ALL, actin layer line.

The muscle biopsy specimens from five young and seven old subjects and more than 1000 membrane-permeabilized fibers were isolated and mounted for small-angle X-ray experiments. The intensities of one myosin meridional reflection (first MM) and of two actin layer lines (sixth and seventh ALLs) were evaluated in the resting state (pCa 9.0) and upon maximal activation, at saturating Ca^2+^ level (pCa 4.5). These intensities were all enhanced at pCa 4.5*,* but the intensities were less pronounced in the old individuals (Table1, Fig.2). To verify that the phenomenon was not due to a compromised filament spacing, the 1, 0 equatorial reflections were evaluated and converted to d1, 0 lattice spacing's, using Bragg's Law (Colson *et al*., 2007). The d1, 0 lattice spacings were not affected by subject age, but the greater widths of the 1, 0 equatorial reflections (estimated via Δ1, 0) suggest a more disordered arrangement in elderly individuals (Table1).

**Table 1:** Different features of X-ray diffraction of muscle fiber in young and old human muscle

	1st MM	6th ALL	7th ALL	1, 0 spacing (nm)	Δ1, 0 (×10^−4^ nm^−1^)
Young relax	0.2831	1	0.2942	41.722	4.465
Young pCa 4.50	0.5891	1.1825	0.3524	39.583	5.059
Old relax	0.1676	1	0.2778	41.634	5.804
Old pCa 4.50	0.3845	1.1233	0.3085	39.544	6.291

### Aging-specific post-translational modifications of different myosin isoforms

**Figure 3:**
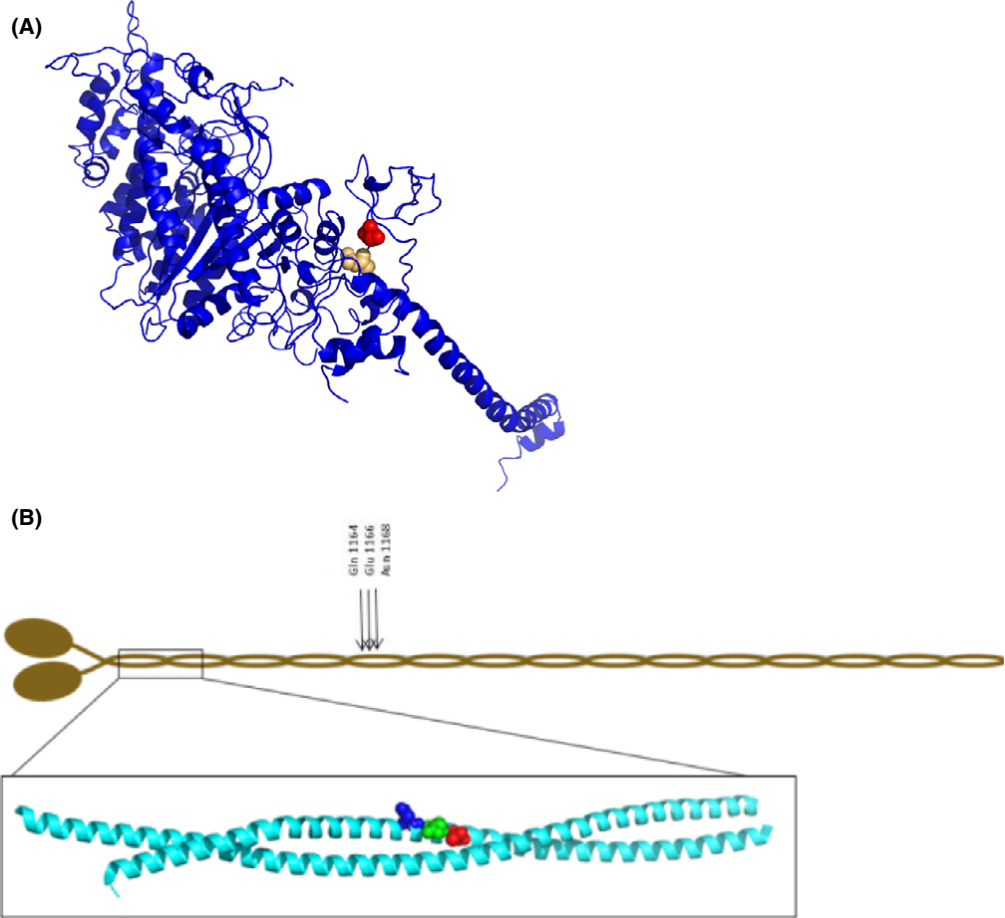
*Myosin post-translational modifications* (A) Ribbon diagram of the molecular motor protein myosin. Two aging-specific post-translational modifications (PTMs) observed in the type IIx MyHC in old human muscle are highlighted in red (carbonylation of Pro79) and in beige (carbonylation of Asn81). (B) Post-translational aging-related modifications in the rod region of all MyHC isoforms (types I, IIa and IIx). A schematic overview of the positions of the amino acids with a specific PTM: deaminations of Gln1164 and Asn1168 and methylation of Glu1166. The positions of the carbonylations at Asp 900 (Blue) Asp904 (Green) and Arg908 (Red) in the coiled-coil structure of the myosin rod are shown in the inset. The images were created with PyMol (http://www.pymol.org) using the atomic coordinates of Protein Data Bank entry 2FXM (Blankenfeldt *et al*. 2006).

A total of five young and nine old human samples from the vastus lateralis muscle were run on 6% SDS-PAGE gel for MyHC isoforms separations. Type I, IIa, and IIx MyHC isoforms gel bands were excised and screened for acetylation, carbonylation, deamidation, glucosylation, methylation, nitration, ubiquitination, and phosphorylation. Eight aging-specific PTMs were identified. Two PTMs, that is, carbonylation of Pro79/Asn81, were observed in the Src-homology domain 3 (SH3) of the myosin motor domain (Fig.3A). However, these PTMs were only observed in the type IIx MyHC isoforms. The other six PTMs, that is, carbonylation of Asp900/Asp904/Arg908, methylation of Glu1166, and deamidation of Gln1164/Asn1168 were all in the rod domain of the myosin molecule and present in all three MyHC isoforms (Fig.3B).

## Discussion

The results from this study confirmed and extended our previous studies: (i) The significant difference between different myosin isoforms in speed and force-generating capacity was confirmed at the motor protein level (Hook *et al*., 1999; Hook & Larsson, 2000; Hook *et al*., 2001; Li *et al*., 2006; Li & Larsson, 2010). (ii) An aging-related slowing of motility speed was observed in both type I and IIa MyHC assays, but the force-generating capacity was unaffected by age, irrespective of MyHC isoform. Similar changes in myosin function were observed in response to *in vitro* oxidative stress of myosin from young individuals. (iii) A myosin molecule arrangement disorder was observed according to X-ray diffraction analyses in old age. (iv) Aging-specific myosin modifications were observed in the rod region of all MyHC isoforms expressed in human adult limb muscles, that is, carbonylations, methylations, and deamidations. It is proposed that these PTMs contribute to the disordered myosin molecule organization and the slowing of motility speed.

Post-translational modifications were observed at identical positions in the myosin rod domain in all MyHC isoforms expressed in adult human skeletal muscle as well as modifications in the S1 globular domain in the IIx myosin isoform. Significantly less is known about the mechanisms underlying the impaired muscle function induced by modifications or mutations in the α-helical coiled-coil rod domain of the myosin molecule than in the globular S1 head region containing the catalytic domain and the actin-binding site. In this study, six-specific aging-related PTMs (carbonylation, methylation, and deamidation) were observed at identical positions in type I, IIa, and IIx MyHC isoforms. The rod domain is essential for the packing of myosin molecules into myosin filaments, for the binding of myosin-associated proteins, and mutations in the rod region result in myopathic phenotypes (Ochala & Larsson, 2012). Perturbations in the rod domain have been shown to disturb sarcomeric uniformity and consequently influence the regulation of muscle contraction (Cammarato *et al*., 2011). This is supported by the disordered myosin orientation in old age according to X-ray diffraction analyses. X-ray diffraction measurements in isolated membrane permeable muscle fibers allow for the detection of structural alterations of contractile proteins with high temporal and spatial resolution (Koubassova & Tsaturyan, 2011). The 1st MM, corresponding to the meridional reflection at 14.5 nm, gives an estimate of the average orientation of the myosin cross-bridges with respect to the thick filament backbone. The 6th and 7th ALLs stand for the actin layer lines at 5.9 and 5.1 nm, respectively and reflect the conformational changes occurring at the actin monomer level (Yagi & Matsubara, 1988). In the present study, as the first MM, sixth, and seventh ALLs were less intensified upon activation in fibers from the older individuals, we suggest that the mean orientation of the myosin heads bound to actin monomers gets more disordered during contraction, inducing an incomplete actin monomer activation and abnormal cross-bridge formation. The d1, 0 lattice spacings were not different between fibers from young and old individuals, but the widths of the 1, 0 equatorial reflections were significantly greater in fibers from the older individuals. These data suggest that the filament spacing is not affected during the aging process but the extent of actin–myosin lattice ordering is altered, leading to a disrupted actomyosin interaction during muscle contraction.

Oxidative stress-induced carbonylation is known to be a common PTM in aging muscle, which was also verified in the myosin molecule in this study. The increased oxidative stress in old age is related to: (i) an increased electron flux and leakage from the mitochondrial electron transport chain during contraction, (ii) high concentration of myoglobin, known to confer greater sensitivity to free radical-induced damage (Thompson, 2009), and (iii) accumulation of reactive oxygen and nitrogen species from oxidative metabolism (Baraibar *et al*., 2013). Two of the five PTMs in the rod region were carbonylations, and the PTMs of Pro79 and Asn81 in the IIx MyHC SH3 domain were carbonylations. Although the exact function of the SH3 domain is not fully understood, the SH3 domain interacts directly with the N-terminal extension of the myosin essential light chain. Essential light chains are crucial for proper myosin functioning (Lowey *et al*., 1993; Lowey *et al*., 2007), influencing ATPase kinetics (Hayashibara & Miyanishi, 1994) and subsequently modulating maximum velocity of unloaded shortening (Greaser *et al*., 1988; Lowey *et al*., 1993). Thus, the IIx MyHC-specific PTMs may have an additive effect on myosin function to the PTMs in the rod region.

The hydrogen peroxide (H_2_O_2_) has been widely used to induce oxidative stress in cellular systems *in vitro*, signified by increased carbonyl content, an indicator of protein oxidation (Dargelos *et al*., 2010; Baraibar *et al*., 2011). In the present study, after treatment with H_2_O_2_, the motility speed of myosin decreased dramatically, which is in agreement with results in aged subjects, implying the oxidative stress-induced modifications compromise the myosin function.

An aging-related decline in maximum force normalized to cross-sectional area (specific force) has been repeatedly documented in membrane-permeabilized single muscle fibers from both humans and rodents (Li & Larsson, 1996; Larsson *et al*., 1997; D'Antona *et al*., 2003; Ochala *et al*., 2006). In this preparation, the excitation–contraction coupling has been bypassed and the loss in specific force is exclusively related to aging-related changes in contractile proteins, but does not exclude additional aging-related changes in regulation of muscle contraction upstream of the contractile protein level. Results from this study at the motor protein level show that the decreased-specific force in old age in membrane-permeabilized muscle fibers is not primarily related to an aging-related change in myosin structure and function. This does not, however, exclude that PTMs in other contractile proteins, such as thin filament proteins, play an important role for the impaired force generating capacity at the single muscle fiber level. In addition, the altered stoichiometric relationship between myosin and actin contents observed in old age in both rodent and human skeletal muscle with a lower myosin to actin ratio in old age represents another potential mechanism contributing to the aging-related decline in specific force (Thompson *et al*., 2006; Cristea *et al*., 2010).

In conclusion, in the present study, we provide for the first time a precise characterization of myosin structural and functional alterations in old age from human muscle. Specific myosin PTMs appear in old age, and they are located in both the myosin motor and rod domains and are likely to contribute to myosin disorder and dysfunction. Such findings are important for the design of potential future ‘preventative’ or ‘therapeutic’ interventions aiming at reversing the muscle weakness in elderly individuals. Successful profiling of aging-specific PTMs may also promote the standardization of sarcopenia diagnosis.

## Experimental procedures

### Subjects

Skeletal muscle tissue from the tibialis anterior and vastus lateralis muscles was collected from 12 young (20–35 years, the median age is 26 years) and 18 old (71–91 years, the median age is 76.5 years) male and female healthy adults. All subjects included in *in vitro* motility assay and mass spectrometry examination were males, and both male and female subjects were included in the X-ray diffraction examinations. All subjects were free of any musculoskeletal disease that may alter muscle function. The study was approved by the ethical committee of the Uppsala University and carried out according to the guidelines of the *Declaration of Helsinki*. Written informed consent was obtained from all subjects enrolled in the present study after they were fully informed of the aim for the experiments and of the risks involved in the biopsy procedure.

### Muscle biopsies and fiber preparation

The muscle samples were obtained using the percutaneous conchotome method from the vastus lateralis and tibialis anterior (TA). Then, samples were separated into two portions. One piece was directly snap-frozen and stored at −80 °C. The other portion was dissected into bundles of approximately 50 fibers in relaxing solution at 4 °C and tied to glass capillaries, stretched to about 110% of their resting slack length. The bundles were chemically skinned by treatment for 24 h at 4 °C in a relaxing solution containing 50% (vol/vol) glycerol and then stored at −20 °C. Within 1 week after skinning, the bundles were cryo-protected by transferring, at 30-min intervals, to relaxing solutions containing increasing concentrations of sucrose (0, 0.5, 1.0, 1.5, and 2.0 m) and then freezing in liquid propane chilled with liquid nitrogen. The frozen bundles were stored at −80 °C. The day before an experiment, a bundle was transferred to a 2.0 m sucrose solution for 30 min and then incubated in solutions of decreasing sucrose concentration (1.5–0.5 m). The bundle was then stored in skinning solution at −20 °C for up to 4 weeks.

### Modified single-fiber myosin *in vitro* motility assay for speed and force measurement

Unregulated actin was purified from rabbit skeletal muscle (Pardee & Spudich, 1982) and fluorescently labeled with rhodamine-phalloidin (Invitrogen, USA). The modified single-fiber myosin *in vitro* motility system for speed and force measurement has been described in detail elsewhere (Hook *et al*., 1999; Hook & Larsson, 2000; Li & Larsson, 2010). In brief, a short muscle fiber segment (1–2 mm) is placed on a glass slide between two strips of grease, and a coverslip placed on top, creating a flow cell of ∽2 μL. Myosin is extracted from the fiber segment through addition of a high-salt buffer (0.5 m KCl, 25 mm HEPES, 4 mm MgCl_2_, 4 mm EGTA and the pH value was adjusted to 7.6 before adding 2 mm ATP and 1% β-mercaptoethanol). After 30 min incubation on ice, a low-salt buffer (25 mm KCl, 25 mm HEPES, 4 mm MgCl_2_, 1 mm EGTA and the pH value was adjusted to 7.6 before adding 1% β-mercaptoethanol) is applied, followed by BSA (1 mg mL^−1^). Nonfunctional myosin molecules were blocked with fragmentized F-actin, and rhodamine-phalloidin labeled actin filaments were subsequently infused into the flow cell, followed by motility buffer to initiate the movement (2 mm ATP, 0.1 mg mL^−1^ glucose oxidase, 23 μg mL^−1^ catalase, 2.5 mg mL^−1^ glucose, and 0.4% methyl cellulose in low-salt buffer). The pH of the buffers was adjusted with KOH, and the final ionic strength of the motility buffer was 71 mm. The flow cell was placed on the stage of an inverted epifluorescence microscope (model IX 70; Olympus America Inc., NY) and thermostatically controlled at 25 °C. Actin movements were filmed with an image-intensified SIT camera (SIT 66; DAGE-MIT Inc., USA) and recorded on videotape.

The unit concentration (0.1 μg mL^−1^ in this study) of α-actinin (A9776; Sigma-Aldrich, Sweden AB, Stockholm) dissolved in the low-salt buffer was chosen as the lowest concentration that would reduce the number of moving actin filaments. Multiples of this concentration were added to the flow cell over a fourfold range in the α-actinin concentration, that is, from 0.1 to 0.4 μg mL^−1^. A specific area on the glass slide was defined by a UV-marking pen (Chr Winther-Sörensen AB, Knäred, Sweden) before creating the flow cell. After identifying an organized moving filament, 3 μL α-actinin was applied to the flow cell, incubated for 1 min, and followed by motility buffer. Moving filaments within the defined area, with constant myosin concentration, were recorded for 12 s to allow measurement of the number and speed of actin filaments at the different α-actinin concentrations, that is, the duration was long enough for reliable measurements, but short enough to minimize the risk of photo-bleaching interference.

For speed analysis, from each single-fiber preparation, 20 actin filaments that were moving at constant speed in an oriented motion were selected. Recordings and analysis were only performed from preparations in which > 90% of the filaments moved bi-directionally. A filament was tracked from the center of mass, and the speed was calculated from ten frames at an acquisition rate of five or one frame (s) per second, depending on the fiber type, using an image analysis package (Image-pro Plus Version 6.0; Media Cybernetics, USA) (Hook & Larsson, 2000). The mean speed of the 20 filaments was calculated. Two force indices were used. First, the slope (regression coefficient, force index_slope_) of the negative linear relationship between the relative fraction of moving filaments and alpha-actinin concentration, that is, the number of moving filaments normalized to moving filaments prior to addition of alpha-actinin vs. increasing alpha-actinin concentrations (the initial recording prior to adding alpha-actinin was omitted from the regression analyses as it may have a disproportional large impact on the regression line). Second, the *x*-axis intercept value of the regression line was also used as a force index (force index_intercept_) (Li & Larsson, 2010).

### Hydrogen peroxide-induced oxidative stress on single muscle fiber

A 4- and 5-mm muscle fiber segment was dissected and placed in relaxing solution at 4 °C, and then, it was cut into two segments with equal length. One segment was incubated with 5 mm Hydrogen Peroxide (H_2_O_2_, 34.01 g mol^−1^; Sigma-Aldrich, Sweden AB, Stockholm) in relaxing solution for 5 min, while the other half was incubated in relaxing solution for the same duration. Both segments were then transferred to relaxing solution for 1 min prior to the *in vitro* motility measurements (described above).

#### X-ray diffraction recordings and analyses

Two to 3 days prior to X-ray recordings, bundles were desucrosed, transferred to a relaxing solution and fibers were dissected. Arrays of approximately 30 fibers were set up (Iwamoto *et al*., 2001; Iwamoto *et al*., 2002; Iwamoto *et al*., 2003; Iwamoto, 2009; Ochala *et al*., 2011). For each fiber, both ends were clamped to half-split gold meshes for electron microscopy (width, 3 mm), which had been glued to precision-machined ceramic chips (width, 3 mm) designed to fit to a specimen chamber. The arrays were then transferred to the skinning solution and stored at −20 °C.

On the day of X-ray recordings, arrays were placed in a plastic dish containing a pre-activating solution and washed thoroughly to remove the glycerol. They were then transferred to the specimen chamber, capable of manual length adjustment and force measurement (force transducer, AE801; Memscap, Bernin, France), and filled with the pre-activating solution. Mean sarcomere length was measured and set to 2.70 μm. Subsequently, X-ray diffraction patterns were recorded at 15 °C, first in the pre-activating solution and then in the activating solution (pCa 4.5) when maximal steady -state isometric force was reached. It should be mentioned that the activating solution was supplied to the chamber using a remote-controlled pump.

For each array, approximately 10–20 diffraction patterns were recorded for each solution at the BL45XU beamline of SPring-8 and were analyzed as described previously (Iwamoto *et al*., 2001, 2002, 2003; Iwamoto, 2009). The wavelength was 0.09 nm, and the specimen-to-detector distance was 2 m. As a detector, a cooled CCD camera (C4880, Hamamatsu Photonics; 1000 × 1018 pixels) was used in combination with an image intensifier (VP5445; Hamamatsu Photonics, Hamamatsu, Japan). To minimize radiation damage, the exposure time was kept low (2 s) and the specimen chamber was moved by 100 μm after each exposure. Moreover, we placed an aluminum plate (thickness 0.35–0.5 mm) upstream of the specimen chamber. The beam flux was estimated to be between 2.7 × 10^11^ and 4.0 × 10^11^ photons s^−1^ after attenuation, and the beam size at the sample position was 0.2 mm (vertical) and 0.3 mm (horizontal). Following X-ray recordings, background scattering was subtracted, and reflection intensities (except for equatorial reflections) were determined as described elsewhere previously (Iwamoto *et al*., 2001, 2002, 2003; Iwamoto, 2009).

Relaxing and activating solutions contained 4 mm Mg-ATP, 1 mm free Mg^2+^, 20 mm imidazole, 7 mm EGTA, 14.5 mm creatine phosphate, 324 U mL^−1^ creatine phosphokinase, 1000 U mL^−1^ catalase, and KCl to adjust the ionic strength to 180 mm and pH to 7.0. Dithiothreitol (DTT) was also added. The pre-activating solution was identical to the relaxing solution except that the EGTA concentration was reduced to 0.5 mm. The concentrations of free Ca^2+^ were 10^−9.0^ m (pCa 9.0, relaxing and pre-activating solutions) and 10^−4.5^ m (pCa 4.5, activating solution).

### Post-translational modifications

Cross-sections from the vastus lateralis muscle of both young and old human subjects were run on 6% SDS-PAGE gels for MyHC isoforms separations. Gel bands corresponding to MyHC I, IIa, and IIx were excised. Gel pieces were destained by multiple rinsing with 50:50 ACN:100 mm NH_4_HCO_3_ followed by reduction with 15 mm solution of dithiothreitol in 100 mm NH_4_HCO_3_ (30 min at 56 °C) and alkylation with 2.1-fold of iodoacetamide (30 min in the dark at room temperature). The liquid was removed, and gel pieces were washed with 100% ACN and then dried at 50 °C. Trypsin solution (25 μg mL^−1^ in 50 mm NH_4_HCO_3_) was added, and the samples were first soaked at 8 °C for 60 min and then digested at 37 °C overnight. After digestion, the samples were sonicated for 5 min and the liquid was collected and dried under vacuum.

A 7-tesla LTQ-FT ICR Ultra tandem mass spectrometer (ThermoFisher ScientificInc. Waltham, MA, USA) modified with a nano electrospray ion source (Proxeon Biosystems, Roskilde, Denmark) was used for all analyses. The samples were diluted in water/TFA (1:0.005 v/v), and peptides were separated by an Agilent 1100 nanoflow system (Agilent Technologies, Santa Clara, CA, USA) equipped with a homemade 15-cm fused silica emitter packed with Reprosil-Pur C18-AQ 3 μm resin (Dr. Maisch GmbH) and coupled online to the mass spectrometer. Peptides were eluted with a 40-min linear gradient from 2% to 45% acetonitrile at 200 nL min^−1^. The mass spectrometer was operated in positive ion mode, and during each run, it automatically switched between a high-resolution (resolving power 50 000) survey mass spectrum in the FTMS cell and consecutive low-resolution CID spectra of the five most abundant ions in the ion trap. The acquired data (.RAW-files) were converted by in-house written program to Mascot generic format files. Peptide identification was performed using the Mascot search engine (version 2.1.3; Matrix Science Ltd, London, UK) by searching the Uniprot-Swissprot database with set ‘Homo sapiens’ taxonomy. Searches were performed with trypsin specificity and up to two missed cleavages were allowed. Mass deviation for precursor ions was set to 0.02 Da. For fragment ions, the mass deviation was set to 0.7 Da.

Carbamidomethylation was chosen as a fixed modification, and the instrument setting was ‘ESI-FTICR’. The peptide/protein identifications were based on MudPIT scoring. To search all possible post-translational modifications, the searches were repeated four times for each sample. The searches were performed with all above mentioned settings and variable modifications set to: (a) deamidation (N, Q), oxidation (M), phosphorylation (S, T), phosphorylation (Y), methylation (C-terminus), and methylation (D, E); (b) deamidation (N, Q), oxidation (M), oxidation (W), oxidation (H), oxidation (R), oxidation (P), oxidation (Y); (c) deamidation (N, Q), oxidation (M), oxidation (D), oxidation (F), oxidation (K), oxidation (N); (d) deamidation (N, Q), oxidation (M), acetylation (K), acetylation (C-terminus), acetylation (C), acetylation (S), nitration (Y), nitration (W).

The output Mascot results were exported as XML files, and data with a peptide scores below 25 were excluded. All remaining peptides we filtered by exclusion of (a) nonmodified peptides; (b) peptides containing only oxidation (M) and/or deamidation (N, Q); (c) peptides belonging to any proteins others than myosin family; (d) same peptides identified multiple times in the same sample.

### MyHC isoforms identification

After motility assay for force and speed measurements, each fiber segment was placed in sodium dodecyl sulfate (SDS) sample buffer in a plastic microfuge tube and stored at −80 °C. The MyHC composition of a muscle fiber was determined by 6% SDS-PAGE. The acrylamide concentration was 4% (wt/vol) in the stacking gel and 6% in the running gel, and the gel matrix included 30% glycerol. Sample loads were kept small (equivalent to ∽0.05 mm of fiber segment) to improve the resolution of the MyHC bands (type I, IIa, and IIx). Electrophoresis was performed at 120 V for 24 h with a Tris-glycine electrode buffer (pH 8.3) at 15 °C (SE 600 vertical slab gel unit; Hoefer Scientific Instruments, Holliston, MA, USA). The gels were silver-stained and subsequently scanned in a soft laser densitometer (Molecular Dynamics, Sunnyvale, CA, USA) with a high spatial resolution (50 μm pixel spacing) and 4096 optical density levels (Larsson *et al*., 1993). The volume integration function (ImageQuant software version 3.3; Molecular Dynamics) was used to quantify the relative amount of each MyHC isoform when more than one isoform was expressed in the fiber segment.

### Statistics

The mean, standard deviation (SD), and median values were calculated according to standard procedures by the software SigmaPlot 12.0 (Systat Software Inc., CA, USA). Student's *t*-test was used for comparisons between two groups after passing the normality test. Two-way ANOVA was used to test the effect of both age and MyHC isoform. Pearson's product moment correlation was used to evaluate linear relations between the fraction of moving filaments and α-actinin concentration. Differences were considered statistically significant at *P *<* *0.05.
